# Vesicular stomatitis virus nucleocapsids diffuse through cytoplasm by hopping from trap to trap in random directions

**DOI:** 10.1038/s41598-020-66942-6

**Published:** 2020-06-30

**Authors:** George Holzwarth, Arnav Bhandari, Lucas Tommervik, Jed C. Macosko, David A. Ornelles, Douglas S. Lyles

**Affiliations:** 1 Department of Physics, Wake Forest University, Winston-Salem, NC 27109 USA; 2 Department of Computer Science, Wake Forest University, Winston-Salem, NC, USA; 3 Department of Microbiology and Immunology, Wake Forest School of Medicine, Winston-Salem, NC, USA; 4 Department of Biochemistry, Wake Forest School of Medicine, Winston-Salem, NC, USA

**Keywords:** Biophysics, Single-molecule biophysics, Biochemistry, Biophysical chemistry, Microbiology, Virology

## Abstract

Within 2–6 hours after infection by vesicular stomatitis virus (VSV), newly assembled VSV particles are released from the surface of infected cells. In that time, viral ribonucleoprotein (RNP) particles (nucleocapsids) travel from their initial sites of synthesis near the nucleus to the edge of the cell, a distance of 5–10 μm. The hydrodynamic radius of RNP particles (86 nm) precludes simple diffusion through the mesh of cytoskeletal fibers. To reveal the relative importance of different transport mechanisms, movement of GFP-labeled RNP particles in live A549 cells was recorded within 3 to 4 h postinfection at 100 frames/s by fluorescence video microscopy. Analysis of more than 200 RNP particle tracks by Bayesian pattern recognition software found that 3% of particles showed rapid, directional motion at about 1 μm/s, as previously reported. 97% of the RNP particles jiggled within a small, approximately circular area with Gaussian width σ = 0.06 μm. Motion within such “traps” was not directional. Particles stayed in traps for approximately 1 s, then hopped to adjacent traps whose centers were displaced by approximately 0.17 μm. Because hopping occurred much more frequently than directional motion, overall transport of RNP particles was dominated by hopping over the time interval of these experiments.

## Introduction

Movement of cellular components is critical for their functions. However, this movement faces a number of barriers, including the dense network of the cytoskeleton^1^. For particles too large to diffuse in the cytoplasm (hydrodynamic radius> approximately 50 nm)^2^, movements consist of a mixture of seemingly random motions and directional motion driven by molecular motors on microtubules and actin filaments^3^. Viruses face the same hurdles during their intracellular replication cycles and use the same cellular mechanisms to distribute viral components throughout the cell. The purpose of the experiments presented here was to determine the transport modes of the ribonucleoprotein (RNP) core (nucleocapsid) of vesicular stomatitis virus (VSV) across the cytoplasm using a variational Bayesian approach to analyze single particle tracks in living cells^4^.

Vesiculovirus Indiana, commonly referred to as VSV (Indiana serotype), is one of the prototypes for the large group of viruses with nonsegmented negative strand RNA genomes (order *Mononegavirales)*. These viruses include many human pathogens, such as measles, Ebola, and rabies viruses, as well as many other viruses that infect both animals and plants. These viruses encode an RNA-dependent RNA polymerase that is responsible for transcription of viral mRNAs and replication of genome RNA, and for most of these viruses, the replication cycle occurs entirely in the cytoplasm^5^. The VSV RNP core consists of the 11 kb RNA genome encapsidated by approximately 1200 copies of the viral N protein. The RNP also contains approximately 400 copies of the viral P protein and 50 copies of the L protein, which together are responsible for the RNA polymerase activity^6^. The resultant mass of the nucleocapsid is 87.1 MDa^7^. Only encapsidated RNA (i.e. RNP) can serve as a template for synthesis of both mRNA and genome RNA. In addition to serving as templates for RNA synthesis, RNPs containing progeny genomes are incorporated into new virus particles by budding from host membranes. Thus, VSV RNPs serve multiple functions and are distributed throughout the cytoplasm of infected cells^8,9^.

VSV RNPs are located near the cell nucleus at the beginning of infection and redistribute during the next 2 to 6 h toward the edges of the cell^9^. During this time the RNPs form aggregates or condensates called inclusion bodies that resemble liquid phase separations, similar to other nonmembranous cellular elements such as nucleoli and P bodies^10^. There is no single transport system for VSV RNPs in infected cells. Both microtubules and actin filaments are involved in distributing VSV RNPs in the cytoplasm^8,9^. Like other cellular particles and organelles, VSV RNPs undergo both seemingly random motions and directional motion driven by molecular motors^8,9,11^. VSV RNP is too large to diffuse freely over long distances in actin-rich areas of the cytoplasm, which have an exclusion limit of approximately 50 nm^2^. When not condensed by M protein, the RNP is a loosely coiled flexible structure with a total length of approximately 3.5 μm^12^. Static and dynamic light scattering analyses of the VSV RNP have determined a hydrodynamic (Stokes) radius (R_h_) of 86 nm and a radius of gyration (R_g_) of around 160 nm at physiologic ionic strength^7^.

Single particle tracking in living cells can be a powerful approach to distinguish different types of particle motion. However, individual particles can undergo multiple types of motion during a single observation lasting 1–10 s. Several methods have been developed to detect different types of motion within particle tracks (e.g.,^4,13–15^). In the experiments described here, we used Bayesian pattern recognition and machine learning methods to divide a track into subtracks. Each subtrack was then tested by mean squared displacement (MSD) analysis, which often reveals the physical mechanism of motion during that subtrack more clearly than does MSD of the entire track. True Brownian motion was not observed during any of the 223 tracks studied. Instead, RNP particles followed one of two physical mechanisms. In the most frequent type of movement, the RNP particle bounced from side to side in a trap or cage. Roughly once per second, the particle jumped to a nearby trap. During the 4 s observation window of this study, particles occupied 1–6 traps differing in their locations by approximately 0.2 μm. The second type of movement, which is much less common, is a driven state in which the particle moves steadily in a preferred direction. This type of movement is probably driven along actin fibers or microtubules by molecular motors. Over the time course of these experiments, overall transport of RNP particles was dominated by hopping. Over longer times, the relative importance of driven motion might increase, if it is always in the same direction, since the average distance traveled is proportional to the square root of the time for movement in random directions, but is proportional to time for directed motion with constant velocity. However, our limited data suggests that driven motion is sometimes directed away from the nucleus and sometimes directed toward the nucleus, undercutting this argument.

## Results

RNP particles were imaged in cells infected with recombinant VSV containing a gene for enhanced green fluorescent protein (eGFP) fused in frame with the P gene (VSV-P-eGFP)^8^. The synthesis of viral RNAs and proteins in cells infected with this virus are similar to those of viruses with wild-type P protein, although the infectious virus produced has an approximately 10-fold lower titer, likely due to less efficient incorporation of the P-eGFP fusion protein into virus particles (approximately 50%)^8^. The distribution in the cytoplasm of infected cells of viral RNP labeled with P-eGFP fusion protein is similar to that of RNP of wild-type VSV labeled with antibody against the nucleocapsid (N) protein^8,9^.

**Figure 1:**
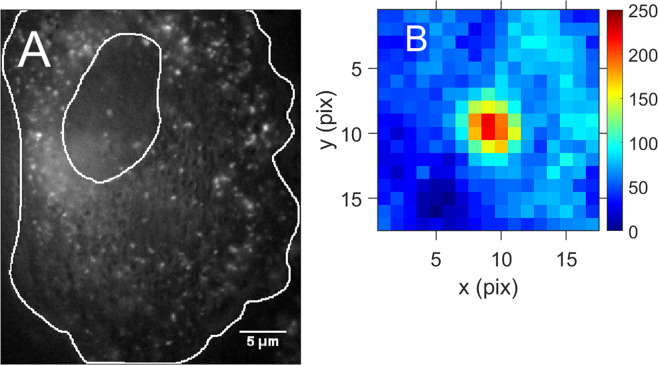
Images of a live A549 cell and a single rnp particle 3 hours after infection by VSV-P-GFP. (**A**) Single frame showing one cell. The small spots are fluorescent RNP particles. The white lines mark the edges of the cell and the nucleus. (**B**) Expanded image of a single rnp particle which stayed in one trap near the nucleus for 4 s. Individual pixels are seen at this magnification. The expanded image is an average of the first 32 frames to improve signal-to-noise, with flat background subtracted. The spot diameter is determined by the point spread function of the objective, not by the (smaller) size of the rnp particle.

Human A549 cells were infected with VSV-P-eGFP virions at a multiplicity of 3 pfu/cell. Live cells were imaged between 3 h and 4 h postinfection. At this time, cells contain many small fluorescent spots, which are primarily individual RNP particles. An image of such a cell is shown in Fig. 1. This time period was chosen in order to be able to analyze the transport of single RNP particles. At later times, the particles aggregate into larger inclusion bodies^9^. The small particles jiggle slightly in random directions when observed by eye (see Supplemental movie). Occasionally a particle moves in a single direction for 0.2 s to 1 s. Images like Fig. 1 were digitized by a scientific-grade CMOS camera operating at 100 frames/s. Stacks containing 400 images were saved for post-processing.

Particles to be tracked were selected visually and tracked by Video Spot Tracker software (CISMM, UNC-CH). Only the smallest particles which remained in focus for all 400 frames were tracked. The tracker can locate particles with subpixel accuracy. Over 200 particles were tracked in 32 cells.

The novel hypothesis of this paper is that the dominant type of movement of the RNP particle is bouncing from side to side in a trap or cage and approximately once per second, jumping to a nearby trap. To objectively detect these states and transitions between them, we constructed mixture models with 1 to 6 two-dimensional Gaussians to fit the 400-frame image stacks. Bayesian statistical methods were used to optimize the parameters of each model and to determine which model had the highest probability of fitting the data. A first-order hidden Markov model was used with the best mixture model to detect the transitions of the system from one state to another and to assign the most probable state to each frame. The transitions between states occur on the timescale of 50 ms − 4 s in these experiments. This allows the 4 s track to be divided into 1–10 shorter “bouts” during which the particle is in a single state. This approach is called pattern recognition and machine learning in the computer science literature^16,17^.

Once a track has been divided into bouts, a log-log plot of the mean-squared-displacement (MSD) versus time delay (τ) between frames in that bout reveals whether the particle is diffusing (slope = 1), or is trapped (slope = 0), or is being driven along a cytoskeletal fiber (slope > 1) at that particular time. No bouts of freely diffusing RNP particles were observed. Instead, each observed bout corresponded either to a spatially localized trap, or to a state in which the particle was driven primarily in a single direction. The fractions of tracks with one to six trapped states, or at least one directionally driven state, are shown in Table 1. The data supporting these results are described in the subsequent sections.

**Table 1:** Distribution of tracks with 1-6 trapped states or at least one directionally driven state.

Number of traps in the track	Number of tracks	Fraction of tracks (*f*_*K*_ or *f*_*driven*_)	Mean Displacement per track (*d* (μm))
1	7	0.03	0.00
2	14	0.06	0.25
3	33	0.15	0.34
4	28	0.13	0.53
5	58	0.26	0.95
6	76	0.34	1.14
directionally driven tracks	7	0.03	1.17
Total	223	1.00	

### Tracks with 1 state

**Figure 2:**
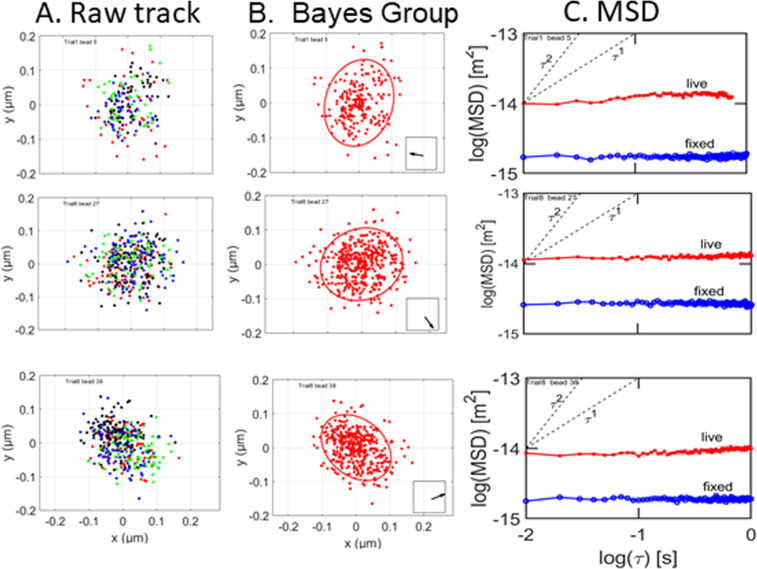
Particles which occupy a single state. Column A: raw tracks. Red symbol: frames1:100; green:101:200; blue: 201:300; black: 301:400. Column B: most probable model found by variational Bayesian analysis. Red symbols: first (and only) state. The ellipses mark the 2σ limits of the fitted Gaussians. The arrow in the box in the lower right corner points from the approximate center of the cell to the particle. Column C, red line: log-log plot of the MSD for an RNP particle in a live cell. Blue line: log-log plot of the MSD of an RNP particle in a fixed cell.

For 3% of the tracks, Bayesian analysis showed that a Gaussian mixture model with one Gaussian was the most probable model. Three examples of such tracks are shown and analyzed in Fig. 2. The data points are confined to an almost circular domain with a radius of approximately 0.1 µm. The color coding for time in the first column shows that the particles moved randomly within the domain. The second column is the result of Bayesian pattern recognition analysis. The ellipses in the second column mark the 2σ limits of the 2D Gaussian which models the data. These ellipses are almost circular, showing that the RNP particles do not have a directional preference while inside these traps.

The third column in Fig. 2 shows plots of the MSD of particles in live and fixed cells. The small slope of the MSD for RNP particles in a live cell (red line) is the classic signature of a particle moving back and forth within a trap. The net displacement of such particles over 4 s is zero. The blue line is for an RNP particle in a fixed cell. The observed MSD for fixed cells is most likely the result of shot noise^18^. The fixed-cell MSD is 20–25% of the MSD for live cells, so shot noise has been ignored.

### Tracks with 2, 3, or 4 states

**Figure 3:**
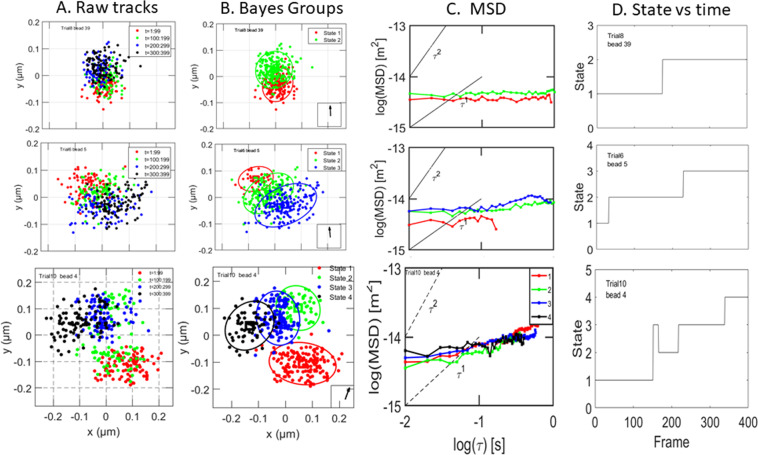
Tracks with 2, 3, and 4 states. Column A: raw tracks. Column B: tracks after Bayesian analysis, showing states detected and ellipses defining Gaussian 2σ limits. Color coding: red is the first state, green is the second state, blue is the third state, and black is the fourth state. Column C: log-log plots of MSD for each state. Column D: state number versus frame number.

About one third of the tracks were best fitted by a model with 2, 3, or 4 states. One example of each is shown in Fig. 3. The raw tracks before Bayes analysis are shown in the first column. The second column shows the same tracks after Bayesian analysis, with color used to distinguish between states.

The first row shows a track with only 2 states, colored red and green. The 2σ boundaries of both states are approximately circular, which shows that motion within each of these traps is not directed. The centers of the two Gaussians in this example are separated by approximately 0.059 μm. A jump from one trap to the next gives net RNP movement which resembles diffusion when viewed in a movie acquired at a slower frame rate. There was not a consistent direction of hopping (see arrows in lower right). The third column shows a log-log plot of MSD vs τ for each state. The MSD’s of both states are almost independent of τ. The fourth column shows the state of the particle as a function of time. The plot shows that there is a single transition between state 1 and state 2 at approximately frame 200. This means that the particle was initially confined by trap 1, then moved to trap 2. Once it moved to the second trap, it did not return to the first one. The likely nature of the traps, and likely mechanisms of escape from the one trap to the next, are considered in the Discussion.

The second row shows data for a particle which occupied 3 traps during the 4 s observation window. There is slightly more overlap between the radii of the ellipses than in the previous case, but the 3 MSD curves again have almost zero slope. The plot in column D shows a single transition between states 1 and 2, and a single transition between states 2 and 3.

The third row shows results for a track with 4 states. There is some overlap between the 4 Gaussians, but each occupies a distinct region within the cytoplasm. All 4 MSD’s have slope near zero. The fourth column shows that the transitions between states are not always irreversible.

Similar results were observed for tracks with 5 and 6 states. Transitions between these states were more frequently reversed.

### Distributions of trap size and trap-to-trap distance

**Figure 4:**
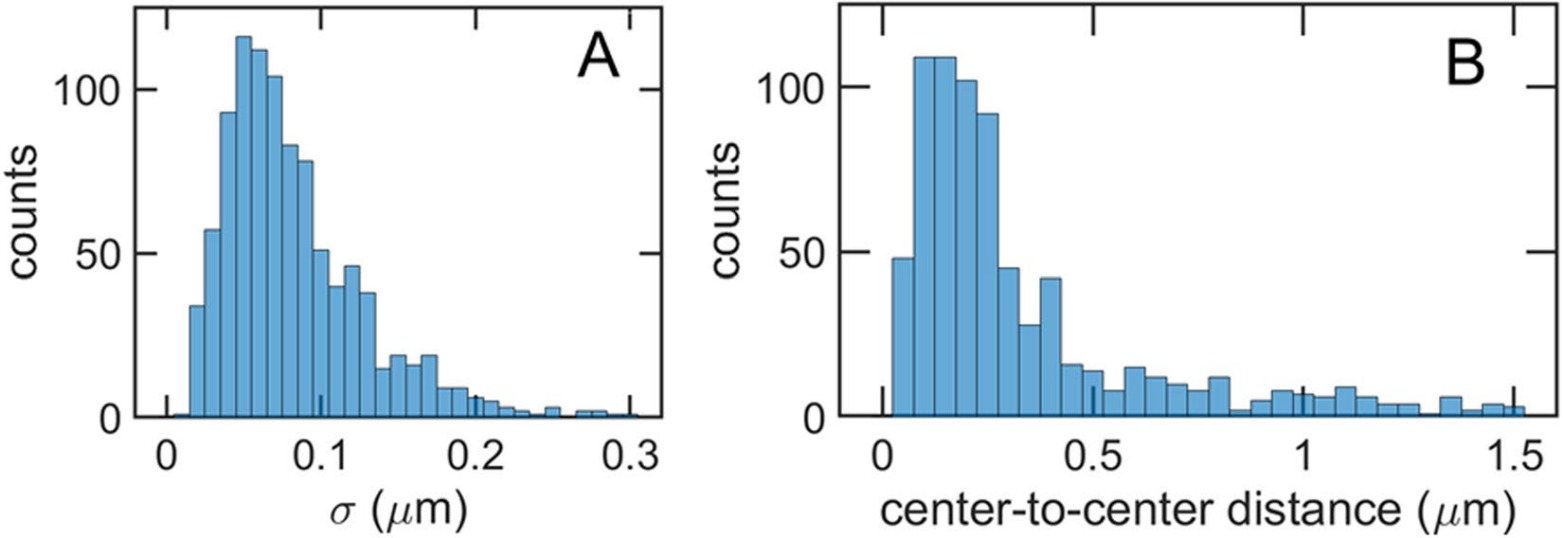
Histograms of trap size and trap-to-trap distance. (**A**) Probability distribution of the width σ = (σ_x_ + σ_y_)/2 of the most probable 2D Gaussians for each trap. The most probable value of σ, estimated from the 3 tallest bins, is approximately 0.060 μm. (**B**) Histogram of the distance between the centers of the traps. Tracks with directed motion are excluded. The most probable value of the center-to-center distance, estimated from the 4 tallest bins, is 0.17  μm. The counts for distances larger than 0.5 μm may be inflated by errors in the sequential numbering of states.

Although single track analysis is emphasized in this manuscript, it is useful to evaluate the global distributions of trap size and trap-to-trap distance for VSV RNP particle transport. Figure 4 shows histograms of these two key parameters for all tracks (approximately 200) except those showing directed motion.

### Tracks showing directional motion

**Figure 5:**
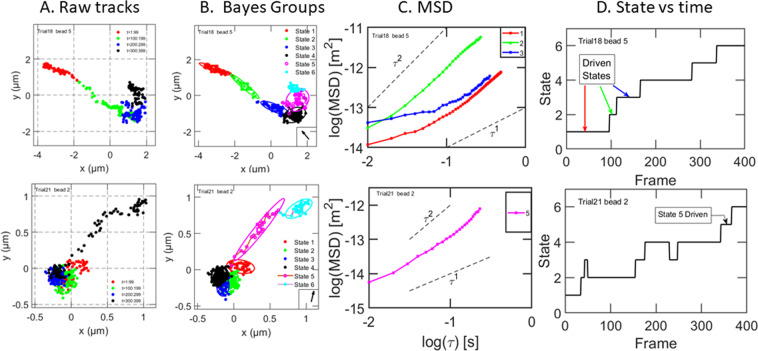
Tracks showing driven motion. Two examples of tracks which are mixtures of trapped and driven states are shown. Column A: raw tracks. Column B: tracks after Bayesian analysis, showing states detected and ellipses defining by the 2σ limits of the Gaussians. Color coding is the same as Fig. 2. The arrow within a box in the lower right corner points from the center of the cell to the particle. The particle in row 1 is moving toward the center of the cell. The particle in row 2 is moving away from the center of the cell. Column C shows a log-log plot of the MSD for the driven states; the MSDs of trapped states are not shown for clarity. Column D is a plot of state number against frame number. The first example moves cleanly from state 1 to state 6. The second example is more complex; some states are visited more than once.

7 out of 223 tracks showed directional motion during at least 1 state. Directional motion was determined primarily from the eccentricity of the ellipse enclosing the xy data of a single state. If a = the major axis of the ellipse and b = the minor axis of the ellipse, then the ratio a/b, called the eccentricity, is a measure of the directionality of the track. If the particles have no directional preference, the ellipse is a circle and the eccentricity = 1. If the particles prefer to move in one direction with respect to the center point of the Gaussian state, the ellipse becomes elongated in that direction and the eccentricity can rise to 8. All tracks with eccentricity> 2.5 were classified as “directed”. Figure 5 shows two examples of such tracks. Both tracks are a mixture of directed and trapped states. The first example in Fig. 5 has 3 directed states with eccentricities = 7.3, 7.2, and 4. The remaining 3 states have eccentricities averaging to 1.8. The second example has one strongly directed state, with eccentricity = 6.5, and 5 trapped states with an average eccentricity equal to 1.7. Each of the states classified as directed has two additional indicators of directed motion. The first is that the individual xy points inside an ellipse do not randomly populate the ellipse with time. Rather, the xy points fill the ellipse systematically in time from one end of the ellipse to the other. By contrast, the ellipses of trapped states are populated at random locations over time. The second additional indicator of directed behavior is the slope of the log-log plots of the MSD for the directed states, shown in the third column of Fig. 5. The observed slopes are between 1 and 2. Such states are sometimes labelled “superdiffusive”. The observed average velocities of the RNP particles in the two tracks shown in Fig. 5 were 0.75 μm/s and 1.05 μm/s. These are in the range measured for kinesin and myosin motors during *in vitro* motility assays^19,20^. Note that directed motion is sometimes toward the nucleus and sometimes away from the nucleus.

### Relative contributions of hopping and driven motion to the transport of RNP particles

In Table 1, 223 tracks are divided into 7 groups according to the number of trapped states (K = 1 to 6) or possessing 1 or more driven states. The fractions of tracks belonging to each of these groups, designated *f*_*K*_ (K = 1:6) and *f*_*driven*_, are given in column 3 of Table 1. The fourth column in Table 1 gives the average root-mean-square displacements ${d}_{K}=\langle {\sum }_{k=1}^{K-1}\sqrt{{({x}_{k+1}-{x}_{k})}^{2}+{({y}_{k+1}-{y}_{k})}^{2}}\rangle$ for tracks with traps. For K = 1, *d*_*K*_ = 0 because there is no hop. For K = 2 to 6, d_K_ increases monotonically from 0.25 to 1.14 μm (Table 1, Column 4). For tracks with a mixture of driven and trapped states, *d*_*driven*_ was evaluated manually by measuring the end-to-end length of the track, not the center-to-center distances. The average directed displacement over 4 s for 7 tracks was 1.17 μm.

Particle displacement flux Φ_*total*_ arises from hopping and from directed motion. Using the data in Table 1, the relative importance of these two sources of transport can be calculated for the 4 s time interval of our experiments:

1A $${{\boldsymbol{\Phi }}}_{total}={\Phi }_{{\rm{hop}}}+{\Phi }_{{\rm{directed}}}$$

1B $${{\boldsymbol{\Phi }}}_{total}=\mathop{\sum }\limits_{K=1}^{6}{f}_{K}{d}_{K}+{f}_{directed}{d}_{directed}$$

1C $${{\boldsymbol{\Phi }}}_{total}=0.77\,\mu m+0.035\,\mu {\rm{m}}$$

The particle displacement flux arising from hopping is about 20 times the particle displacement flux arising from directed motion, primarily because hopping occurs much more frequently than driven motion. An experimental test over longer times and longer distances would be useful but is not straightforward because of photobleaching.

### A model for RNP particle motion in a harmonic trap

We tested whether the observed movements of trapped RNP particles could be simulated by solutions of the Langevin equation for the thermally driven motions of a sphere immersed in a viscous fluid with added harmonic potential. The Langevin equation then has 4 terms arising from inertia, viscous drag, the potential well, and stochastic solvent collisions^21^:

2 $$\mathop{\underbrace{m\ddot{x}(t)}}\limits_{{\rm{inertia}}}=\mathop{\underbrace{-\,\gamma \dot{x}(t)}}\limits_{\mathop{{\rm{viscous}}}\limits_{{\rm{drag}}}}+\mathop{\underbrace{kx(t)}}\limits_{\mathop{{\rm{force}}\,{\rm{of}}}\limits_{{\rm{trap}}}}+\mathop{\underbrace{\sqrt{2{k}_{B}T\gamma }\,W(t)}}\limits_{\mathop{{\rm{force}}\,{\rm{of}}\,{\rm{solvent}}}\limits_{{\rm{collisions}}}}$$

Here *m* is the particle mass, γ is the viscous drag coefficient of the fluid on the sphere, *k* is the spring constant of the trap, *k*_*B*_ is Boltzmann’s constant, *T* is temperature in Kelvin, and *W(t)* is a stochastic Weiner process with mean = 0 and standard deviation = 1. From Stokes’ Law, the drag coefficient γ is equal to 6πηR, where η is the fluid viscosity, taken as 0.006 Pa·s^1^ and R is the radius of the sphere, taken as 0.086 μm^7^.

**Figure 6:**
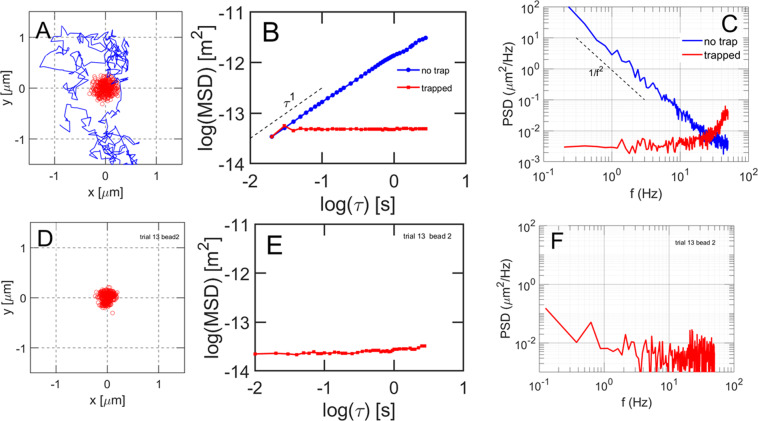
Solutions of the Langevin equation with and without a trap, and comparison to experimental data. (**A**) Blue line: numerical solution of the Langevin equation for a spherical particle undergoing free Brownian motion (no trap) in a viscous medium. Red symbols: simulated xy track for the same particle radius, viscosity, and temperature but with added harmonic potential k_x_ and k_y_ = 1.5E-06 N/m. Both simulations are for 400 steps each of duration 10 ms. (**B**) Log-log plots of the MSD’s of the simulated tracks of trapped and untrapped particles. (**C**) Power spectral density of the simulated tracks shown in panel A. Both curves are an average of 10 simulations to reduce noise. (**D**) Experimental xy track of an RNP particle in a trap. (**E**) Log-log plot of the experimental MSD for the single particle in a trap. (**F**) Power spectral density of the experimental track of the RNP particle in a single trap.

Numerical integration of this stochastic differential equation gives x(t). The second dimension, y(t), is obtained in the same way. The spring constant is adjusted to make the area of the simulated track agree approximately with the experimentally observed track area of particles which stay in one trap during the observation period (Fig. 2). Figure 6 shows the results of the simulations for trapped and untrapped particles and compares the simulations to observed data.

Numerical solutions of the Langevin equation with and without a trap are shown in Fig. 6A. Without a trap, the particle occupies a space of roughly 1 μm x 1  μm. When a potential well with spring constant k_x_ = 1.5×10^−6^ N/m is added to Eq. 2, the area occupied decreases to roughly 0.2μm × 0.2μm, a factor of 1/25 in area. Figure 6B shows a log-log plot of the MSD’s of the two simulated tracks. Figure 6C shows the spectral energy density of these two simulated paths. Free Brownian motion exhibits the expected τ^1^ dependence in the MSD plot and *1/f*^2^ frequency dependence in the power spectral density plot. The spectral energy density of the simulated particle in a trap is largely independent of *f* within the experimental time limits.

An example experimental track for an RNP particle which occupied a single trap is shown in Fig. 6D for comparison to the simulation. By choice of the spring constant k, the area occupied by the simulated particle in the harmonic trap is similar to the area occupied experimentally. The MSD and spectral energy density of the simulation (Fig. 6B,C) then agree well with experiment (Fig. 6E,F) without adjusting additional parameters.

## Discussion

The data presented here show that VSV RNP particles bounce from side to side in a trap or cage and move through the cytoplasm of infected cells primarily by hopping from trap to trap approximately once per second. Motion within the trap is not directional and is consistent with Brownian diffusion within a springlike (harmonic) trap. The trap-to-trap hopping appears to be in random directions, although the data do not rule out an overall direction to the flow of particles that might be apparent upon analysis of a larger number of particles or longer time periods. Very occasionally, RNP particles move briefly in a non-random, directed manner at a velocity of approximately 1 μm/s.

We showed previously that migration of VSV RNPs in VSV-infected cells is dependent on both microtubules and actin filaments^9^. This conclusion was supported by live cell imaging experiments similar in design to the ones presented here. In both studies, a fast CMOS camera allowed image acquisition at 100 frames/s. The greater insights presented here were revealed by two experimental improvements and one change in the analysis of data. The present experiments were performed in human A549 cells instead of HeLa cells, because the A549 cells are somewhat flatter, with a well spread cytoplasm that facilitates analysis of particle movement in two dimensions. Also morphological changes associated with VSV cytopathic effects are slower to develop in these cells relative to other lines such as HeLa cells. The second experimental improvement was that conditions were found under which VSV-infected cells contained mostly single RNP particles rather than aggregates by analyzing cells between 3 and 4 h postinfection. Data analysis was improved by the application of two-dimensional variational Bayesian analysis. This allowed individual particle tracks to be objectively divided into short bouts, or states, during which the particle was in a single trap, rather than the usual procedure of evaluating ensemble averages of properties such as the MSD.

Our previous studies used ensemble averaging to show that on short time scales (0.01 s ≤ τ <approximately 1 s), motion of RNP aggregates was subdiffusive (slope «1), whereas at longer time scales (τ > approximately 1 s), the motion was diffusive (slope ≈ 1).The new results presented here show that the subdiffusive behavior at short τ comprises primarily bouncing within the traps, and the diffusive behavior at τ > 1 s is made up primarily of hops between traps combined with less frequent directed motion.

The experiments presented here were bounded by the constraints of photobleaching and signal-to-noise. Photobleaching, a well-known problem with GFP in live cells, limited stack lengths to 400 images if adequate signal-to-noise was to be achieved at 100 fps. As mentioned above, longer analyses might reveal an overall directionality to the hopping motion. At the other end of the time range is the limitation of time resolution to 10 ms. Data acquisition at shorter exposure times could allow better analysis of the bouncing of RNP particles from side to side in the trap, but fluorescence source intensity would have to be increased inversely with exposure time if signal-to-noise ratio is to be maintained, increasing bleaching.

Future experiments will be to identify cellular elements that constitute the traps, and how those components mediate transport of RNP particles. Cytoskeletal fibers and cellular organelles near the particles are likely candidates for forming the traps. Biochemical interventions, for example by cytoskeletal inhibitors or gene silencing, when combined with single particle tracking, may allow tests of these hypotheses.

## Materials and Methods

### Cells and Virus

A549 human lung adenocarcinoma cells (ATCC CCL-185) were cultured in Dulbecco’s modified Eagle medium containing 7% fetal bovine serum at 37 C and 5% CO_2_. This cell line was chosen for these experiments because the cells are relatively flat, with a well spread cytoplasm that facilitates analysis of particle movement in two dimensions. Also, morphological changes associated with VSV cytopathic effects are slower to develop in these cells relative to other lines such as HeLa cells (unpublished observations). Stocks of recombinant VSV containing a gene for enhanced green fluorescent protein (eGFP) fused in frame with the P gene^8^ were prepared in BHK cells. Cells were infected at a multiplicity of 0.01 plaque-forming-units (pfu) per cell in Dulbecco’s modified Eagle medium containing 2% fetal bovine serum for 24 h. Cell-free supernatants were titered by plaque assay in BHK cells. For experiments, A549 cells were seeded in 35-mm glass-bottom culture dishes (WillCo 70670-52) at 2.5×10^5^ cells per dish. After incubation overnight, cells were infected at a multiplicity of 3 pfu/cell, and live cells were analyzed between 3 and 4 h postinfection. Fixed cells were prepared by incubation for 15 min at room temperature with 4% paraformaldehyde in phosphate-buffered saline (pH 7.0), then mounted in ProLong Gold Antifade Mountant with 4′,6-diamidino-2-phenylindole (DAPI) (ThermoFisher Scientific, catalog number P36931).

### Microscopy

35-mm glass-bottom culture dishes (WillCo 70670-52) containing cells infected with VSV-P-eGFP were placed on the stage of a Nikon Ti inverted microscope fitted with a 60×/NA1.4 oil objective. The microscope was placed on an optical breadboard (Newport) mounted onto 4 pneumatic vibration isolators (Thorlabs PWA075). The stage environment was set to 37 C, 5% CO_2_. The light source for fluorescence microscopy was a metal halide short-arc lamp (Lumen Dynamics X-CITE 120). The epi-illumination cube for GFP consisted of a 470/40 nm excitation filter, 495 nm dichroic filter, and 525/50 emission filter (Chroma 49002). Images were digitized to 16 bits at 100 frames per second by a CMOS camera with 6.5 μm x 6.5 μm pixels (PCO Edge 5.5).

Setting the intensity of the excitation light for fluorescence imaging requires a balance between two competing factors. The first is bleaching, which can be reduced by decreasing the source intensity or the duration of image stacks. The second is signal-to-noise ratio for the RNP particle spots, which arises from photon noise and background fluorescence. Fortunately, each RNP particle contains approximately 400 eGFP groups. This allows the competing needs to be met if the duration of the image stacks is limited to about 4 s when the frame rate is 100 fps.

### Tracking

ImageJ was used to select a subimage of a single cell containing many small, well-isolated RNP particles. Subpixel tracking of these particles was done by Video Spot Tracker software (CISSM-UNC-CH) using cone mode with precision set to 0.001. The subpixel precision of the tracker itself was tested by tracking 400 nm fluorescent beads attached to the surface of a coverslip. The diameter of the bead track was 0.15 pixel (0.016 μm), which is negligible compared to the smallest diameters of RNP tracks for live cells, which are 0.2 μm (Fig. 2).

Photoelectron count noise^18^, also known as shot noise, can affect tracking precision. An upper bound to the effect of shot noise was obtained by comparing the MSD’s of RNP particles in fixed cells to those of live cells. The diameters of fixed cell RNP particle tracks were 0.06 to 0.07 μm, which is less than half the diameter of the smallest live cell RNP tracks (Fig. 2, column A). Log-log plots of the MSD’s of fixed and live-cell RNP particles (Fig. 2, column C) shows that the log of the MSD for fixed cells is −14.7 compared to −14.0 for the log of the live cells. This shows that shot noise is a concern but does not change qualitative conclusions.

### Bayesian pattern recognition

Each particle track is a set of 400 {x,y} values each corresponding to a specific time. The first goal of the analysis was to test the track for clustering in space and time, using Gaussian mixture models with 1 to K Gaussians. K is the number of states (groups or clusters) in the model. Variational Bayesian analysis was used to optimize each Gaussian and to objectively determine which mixture of K Gaussians had the highest probability of fitting the {x,y} data. The second goal was to determine the Gaussian to which each x,y pair most probably belonged. The methods used to reach these goals are now standard techniques of pattern recognition and machine learning^16,17^. Bronson *et al*.'s Matlab package^22^, vbFRET [Source Forge], was used as the starting point for our code. We extended vbFRET from 1D to 2D following Murphy^16^ and Bishop^17^.

The main steps are:

1. Construct a candidate model with K 2D Gaussians, to create a Gaussian Mixture Model (gmm). Add hidden variables z_n_, n = 1:N, N = numFrames, to account for the initially unknown state of the system at each time point. Randomly initialize Θ, the vector of 5 parameters of each Gaussian (amplitude, center_x, center_y, variance_x, variance_y). Compute an initial value for L, the lower bound of the probability that the model fits the data.
2. Adjust Θ using 2 steps of K-means to improve on the initial estimates of model parameters. Data points are assumed to be independent.
3. Construct a first-order hidden Markov Model (HMM) to describe transitions between the hidden states z from frame to frame. Apply a variational Bayes algorithm to this gmm-HMM model to improve Θ and assign posterior probabilities p(z_i_ = k|x_i_, Θ) to each z. The variational update equations are coupled, so updating must be done iteratively over alternating E and M steps:

3.1 E step. Keeping Θ fixed, adjust the responsibilities of each hidden variable z_n_ to improve L. Use the Beal forward-backward algorithm^23^ to account for the time-sequence nature of the data. Recheck L. If it has increased, continue the iteration. If the change in L is less than a predetermined limit (ε,) terminate the iteration.

3.2 M step. Keeping the responsibilities fixed, adjust Θ to improve L. Repeat the E and M steps until the change in L is less than ε.

The result is an optimized set of Gaussian parameters and responsibilities. Use the Viterbi algorithm^24^ to find the hidden state sequence with the highest global probability. This reassigns the hidden variable z of each frame from a set of K responsibilities to a single responsibility (state), which may not be the state with the highest responsibility. Frames in bouts of duration 5 or less were reassigned to their adjacent bouts. This affected about 30% of tracks when K = 5 or 6.

### Mean Squared Displacement (MSD)

MSD is defined, in the usual way, as

3 $$msd(\tau )=\langle {[x(t+\tau )-x(t)]}^{2}+{[y(t+\tau )-y(t)]}^{2}\rangle $$

The values of *τ* range from 0.01 s to 3 s for the experiments in this paper.

### Simulation of Brownian motion

Brownian motion was simulated by numerical integration of the stochastic Langevin equation (Eq. 2). The inertia term was ignored because it has negligible effect at the time scales of our experiments. The update procedure consisted of N iterations, each of duration Δt. Each iteration is comprised of a deterministic step and a diffusive step^21^:

1: A deterministic step involving drift generated by k_x_x, the gradient of the harmonic potential, divided by the friction factor γ:

4 $${\rm{x}}({\rm{i}})={\rm{x}}({\rm{i}}-1)-{{\rm{k}}}_{{\rm{x}}}{\rm{x}}({\rm{i}}-1)\varDelta {\rm{t}}/\gamma $$

2: A diffusive step arising from the random impulses of solvent molecules:

5 $${\rm{x}}({\rm{i}})={\rm{x}}({\rm{i}})+{(2{\rm{D}}\varDelta {\rm{t}})}^{1/2}{\rm{randn}}()$$

The inertia term in Eq. 2 can be ignored because it is small compared to the other terms in Eq. 2. D is the diffusion constant of the particle, assumed to have radius R = the Stokes radius of the particle. The variable “randn” is a normally distributed random number with mean = 0 and variance = 1.

The evaluation of y(i) is done similarly, but independently.

### Power Spectral Density (PSD)

In electrical engineering, the power is proportional to V^2^. We replaced

V^2^ with x^2^. PSD is the power per unit of frequency. The Matlab fft-based code used to compute the power spectral density was:

N = length(x);

xdft = fft(x);

xdft = xdft(1:N/2 + 1);

psdx = (1/(Fs*N)) * abs(xdft).^2;

                             % square the fft to determine power.

psdx(2:end-1) = 2*psdx(2:end-1);

                            % clean up endpoints.

freq = 0:Fs/length(x):Fs/2;

                           % evaluate corresponding frequencies for plot

## Supplementary information

Supplementary information.

Supplementary information 2.

## Data Availability

The datasets generated during and/or analysed during the current study are available from the corresponding author on reasonable request. The Matlab code for the 2D variational Bayesian analysis, named vbTRACK_2D, will be added to the 1D code previously uploaded to SourceForge as “vbTRACK”.
