# Supplementary material for: Vesicular stomatitis virus nucleocapsids diffuse through cytoplasm by hopping from trap to trap in random directions
Source: Sci Rep. 2020 Jun 30;10:10643. doi: 10.1038/s41598-020-66942-6 (PMC7326962; doi:10.1038/s41598-020-66942-6)
Supplement: Supplementary file 1 — Supplementary information. [file 41598_2020_66942_MOESM1_ESM.docx]

Supplementary gif movie of rnp particle motion in live cell at 37C. Movie acquired at 100 fps with Nikon Ti using 60X/oil objective and PCO sCMOS camera.
