# Supplementary figures and images for: Vesicular stomatitis virus nucleocapsids diffuse through cytoplasm by hopping from trap to trap in random directions
Source: Sci Rep. 2020 Jun 30;10:10643. doi: 10.1038/s41598-020-66942-6 (PMC7326962; doi:10.1038/s41598-020-66942-6)

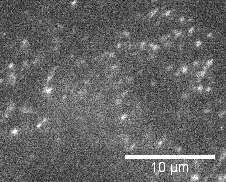

Supplement: Supplementary file 2 — Supplementary information 2. [file 41598_2020_66942_MOESM2_ESM.gif]
